# Long-Term Sequences of Intergenerational Living Arrangements Among Older Americans

**DOI:** 10.1093/geroni/igaa057.359

**Published:** 2020-12-16

**Authors:** BoRin Kim, Jersey Liang, Xiao Xu, Mary Beth Ofstedal, James Raymo

**Affiliations:** 1 University of New Hampshire, Durham, New Hampshire, United States; 2 University of Michigan, Ann Arbor, Michigan, United States; 3 Yale University, New Haven, Connecticut, United States; 4 Princeton University, Princeton, New Jersey, United States

## Abstract

Living arrangements are critical for intra-family exchanges such as physical, financial, and emotional supports influencing older adults’ health and well-being. Existing research is largely based on short-term observations of living arrangements. This study aims to explore longer term dynamic patterns of intergenerational living arrangements among older Americans and their sociodemographic and health determinants. Data came from the 1998-2016 Health and Retirement Study. Sequence analysis was employed to identify long-term patterns of intergenerational living arrangements for 3,025 individuals who were age of 51-64 at the baseline (ages of 69-82 at the last wave), have at least one child, and were observed 10 consecutive times (Obs.=30,250). Living arrangements were categorized into co-residence, proximate residence (i.e., 10miles from children), and nursing home. Multinomial logistic regressions were used to evaluate the associations of individual characteristics with the different living arrangements sequences. Four patterns of eighteen-year living arrangement trajectories were identified: Transition to proximate residence (17%), stable in distant residence (24%), stable in proximate residence (38%), and stable in co-residence (22%). Younger age and working (vs. retired) status were associated with stable coresidence rather than proximate or distant residence. Respondents who retired during the study period were more likely to move close to their children. Contrary to expectations, changes in self-rated health and functional status had no significant effect in long-term living arrangement sequence patterns. These findings suggest that intergenerational living arrangements among older Americans tend to be stable and not to be significantly affected by their caregiving needs.

